# Gene expression pattern analysis using dual-color RT-MLPA and integrative genome-wide association studies of eQTL for tuberculosis suscepitibility

**DOI:** 10.1186/s12931-020-01612-9

**Published:** 2021-01-20

**Authors:** Jing-Wen Ai, Hanyue Zhang, Zumo Zhou, Shanshan Weng, Heqing Huang, Sen Wang, Lingyun Shao, Yan Gao, Jing Wu, Qiaoling Ruan, Feifei Wang, Ning Jiang, Jiazhen Chen, Wenhong Zhang

**Affiliations:** 1 Department of Infectious Diseases, Huashan Hospital, Fudan University, 12 Wulumuqi Zhong Road, Shanghai, 200040 China; 2 State Key Laboratory of Genetic Engineering and Institute of Biostatistics and Computational Biology, School of Life Sciences, Fudan University, 138 Yixueyuan Road, Shanghai, 200032 China; 3 Department of Infectious Diseases, People’s Hospital of Zhuji, 122 Huanshan South Road, Zhuji, 311800 China

**Keywords:** Latent tuberculosis, Active tuberculosis infection, Genetic susceptibility, Single nucleotide polymorphisms, Expression quantitative trait loci

## Abstract

**Background:**

When infected with *Mycobacterium tuberculosis*, only a small proportion of the population will develop active TB, and the role of host genetic factors in different TB infection status was not fully understood.

**Methods:**

Forty-three patients with active tuberculosis and 49 with latent tuberculosis were enrolled in the prospective cohort. Expressing levels of 27 candidate mRNAs, which were previously demonstrated to differentially expressed in latent and active TB, were measured by dual color reverse transcription multiplex ligation dependent probe amplification assay (dcRT-MLPA). Using expression levels of these mRNAs as quantitative traits, associations between expression abundance and genome-wild single nucleotide polymorphisms (SNPs) were calculated. Finally, identified candidate SNPs were further assessed for their associations with TB infection status in a validation cohort with 313 Chinese Han cases.

**Results:**

We identified 9 differentially expressed mRNAs including *il7r, il4, il8, tnfrsf1b, pgm5, ccl19, il2ra, marco* and *fpr1* in the prospective cohort. Through expression quantitative trait loci mapping, we screened out 8 SNPs associated with these mRNAs. Then, CG genotype of the SNP rs62292160 was finally verified to be significantly associated with higher transcription levels of IL4 in LTBI than in TB patients.

**Conclusion:**

We reported that the SNP rs62292160 in Chinese Han population may link to higher expression of *il4* in latent tuberculosis. Our findings provided a new genetic variation locus for further exploration of the mechanisms of TB and a possible target for TB genetic susceptibility studies, which might aid the clinical decision to precision treatment of TB.

## Background

Tuberculosis (TB) remains one of the most important threats to human health for thousands of years. According to a report of the World Health Organization (WHO) in 2017, about 10 million individuals newly infected with TB and 1.3 million died [1]. Most people infected with *Mycobacterium tuberculosis* (*Mtb*) can clear *Mtb.* However, approximately a quarter to three quarters of the population would keep a latent tuberculosis infection (LTBI) status and among them, about 5–10% persons will develop active tuberculosis (ATB) infections within their lifetimes [2].

Although living conditions, age, immune status could be predictors for ATB, with the development of molecule biology, the host genetic factors were thought to be substantial risk factors [3–5]. Kramnik et al. found that susceptibility to *Mtb* was a complex multigenic trait [6]. The study enrolling 202 co-twins suggested that the tuberculosis incidence among identical twins was higher than that among fraternal twins, which provided more evidence that the host genetic factors might affect the susceptibility to active tuberculosis infections [7].

In 2007, researchers firstly analyzed the gene-expression profiles of mRNA in whole blood samples from patients with ATB and LTBI [8]. Studies have shown that the expression levels of the host mRNAs vary at different post-infection status. It has been confirmed in several studies that expression levels of genes such as FcγRIB, FCGR1A, RAB33A, RAS and RIN3 could discriminate between ATB and LTBI patients [8]. Besides, some studies tried to analyze the association between human genetic variations and TB infection phenotype. In 2010, Thy et al. identified a single nucleotide polymorphism (SNP) rs4331426 located in a gene-poor region on chromosome 18q11.2 in African population by genome-wide association study (GWAS), which might make sense in infection of *Mtb *[9]. Then in 2012, they identified another SNP rs2057178 in Ghanaian, Gambian, Indonesian and Russian individuals using the same method [10].

Although GWAS can link SNPs with different disease phenotypes, some identified SNPs might locate on non-coding regions and thus provided limited information for the exploration of the function of the genetic variations. Mapping of expression quantitative trait loci (eQTL) can help to go a further step to associate differential mRNA expression with SNPs, which provides in-depth understanding in studying disease predisposition loci with potential function [11]. Previous studies have already identified SNPs associated with the discriminatory expression levels between *Mtb* infected and uninfected dendritic cells which were derived from peripheral blood mononuclear cells. Barreiro et al. performed eQTL mapping to identify *DUSP14 and* rs712039 as tuberculosis susceptibility genes [12]. Siddle et al. analyzed the expression levels of miRNA using eQTL and identified two SNPs, rs532751 and rs11159250 that could alter the expression of miR-326 and miR-1260[13]. These two studies analyzed the different expression profiles in healthy and infected cells, but they didn’t explore the differentiation of these profiles between ATB and LTBI. Besides, most of the previous studies were in Africa and Europe. Because the genetic backgrounds of different populations vary widely, conclusions from studies of other ethnic groups can not be applied in Chinese.

In our study, we directly analyzed the expression levels of candidate mRNAs in peripheral blood from ATB patients and LTBI patients using dcRT-MLPA, and conducted expression quantitative trait loci. Furthermore, we tried to find the associations between expression abundance and genome-wild single nucleotide polymorphisms (SNPs). Overall, this study aimed to comprehensively analyze the susceptible genes associated with tuberculosis infection and the possible mechanism of TB genetic susceptibility in Chinese Han population.

## Methods

### Ethics approval

This study was approved by ethical committees in Hua Shan Hospital (Shanghai, China) and we had already obtained written informed consent from all study subjects.

### Prospective cohort enrollment

#### Patients and healthy donors

In this study, 92 adults aged 18 to 65 were enrolled in the prospective cohort.

ATB patients and LTBI patients were recruited at Zhuji People's Hospital of Zhejiang Province(Zhejiang, China) and 905th Hospital of PLA Navy (Shanghai, China). Individuals satisfying at least one of the following criteria were enrolled to ATB groups: (1) positive results of *Mtb* culture of respiratory specimens; (2) positive results of acid-fast bacilli smear microscopy; (3) positive histopathological findings together with TB clinical assessment: a history of close contact with ATB patients, positive clinical signs and chest radiography (e.g. cavities, hydrothorax, or changes on serial chest X-rays). All patients were diagnosed as active pulmonary tuberculosis and treated according to the guideline. LTBI patients were individuals who were closely household contact with the diagnosed ATB patients. They all had positive T-SPOT TB test findings and were without any clinical evidence of ATB.

Participants meeting any one of the following criteria were excluded: human immunodeficiency virus (HIV) testing positive, receiving immunosuppressive, chemical or biochemical therapy, treated for ATB for more than 8 weeks, or patients with abnormal liver function (AST, ALT ≥ 2ULN, TB ≥ 2.5 mg/dl).

### Preparation of RNA

Two and half milliliters of whole blood were drawn from each individual in the prospective cohort with PAXgene blood RNA tube (Qiagen, German). According to the manufacturer’s instructions of kit, then we isolated and purified RNA using the PAXgene Blood RNA extraction kit(Qiagen, German). The concentration and the quality of RNA from whole blood were assessed by NanoDrop 2000 spectrophotometer (Thermo Fisher Scientific, USA).

### Whole blood dual color reverse transcription multiplex ligation dependent probe amplification assay (dcRT-MLPA)

After reviewing and summarizing previous related literature and selected out 27 mRNAs as target mRNAs, all of which were reported to be differentially expressed between latent and active tuberculosis patients (*il4, sec14l1, timp2, il7r, ltf, casp8, il8, mmp9, tnfrsf1a, tnfrsf1b, ccr7, ifng, pgm5, bcl2, tgfb1, ccl19, cd4, cd8a, il2ra, il6, foxp3, marco, tnfsf10c, blr1, fpr, fcgr1a* and *cxcl10*) (Additional file 1: Table S1).

We designed immediately half probes for each target-specific sequence and also designed specific RT primers for the probe target sequence. All half probes and RT primers used in this study were described in details in Additional file 1: Table S2.

We conducted reverse transcription using the PrimeScriptTM RT reagent kit (Takara, Japan) and SLASA MLPA-RT RNA reagent kit (MRC-Holland, USA). The reaction conditions were described in Additional file 1: Table S3. Using HiDi formamide-containing 400 HD ROX size standard, the product of PCR amplification was diluted 10 times and then analyzed on 3730 capillary sequence in GeneScan mode (Applied Biosystems, USA). As for trace data analysis, we used the GeneMapper software package (Applied Biosystems, USA). We used B2M as reference gene and calculated the relative expression level of selected mRNAs to compare with B2M expressions.

### DNA extraction and genome-wide SNP assay

For each sample, the genomic DNA was extracted from 200 µl of peripheral venous blood using PAXgene blood DNA tube (Qiagen, German) then extract genomic DNA according to manufacturer’s instructions. DNA content, purity, and integrity were determined using NanoDrop 2000 spectrophotometer (Thermo Fisher Scientific, USA). The genome-wide SNP assay was performed by the Infinium OmniZhongHua-8 BeadChip(Illumina, USA), which could detect 887,270 SNP genotype across the whole genome.

### Mapping of expression quantitative trait loci (eQTLs)

EQTL mapping can associate the transcript abundance with specific genomic. Expression levels are viewed as quantitative traits, and associations between SNPs and expression abundance were calculated using a mixed-linear regression model:

$${\text{gene expression}} = \beta_{1} \times {\text{Genotype}} + \beta_{2} \times {\text{age}} + \beta_{3} \times {\text{gender}} + \beta_{4} \times {\text{kinship}} + \varepsilon$$

where patient age, gender and kinship information were considered as covariances in the above model. Gene expression phenotypes were mapped to particular genomic loci by combining studies of variation in gene expression patterns with genome-wide genotyping. The genome-wide association analysis was executed under Plink (v1.90b4.4 64-bit).

### Statistical analysis

Microsoft excel spreadsheet version 2013 (Microsoft Corp., Redmond, WA, USA) and Microsoft access version 2010 (Microsoft Corp., Redmond, WA, USA) were used for data entry and management. As for the comparison between two groups, if the continuous variable conforms to the normal distribution, the t-test was used, while Mann Whitney U rank sum test was used for the non-normal distribution; the chi-square test or Fisher's exact test was used for the comparison of categorical variables. As for comparison between more than two clinical groups, a non-parametric Kruskal–Wallis test was performed. The statistical significance cut-off level was p < 0.05. All data was analyzed using SPSS 20.0 (IBM Corp., Armonk, NY, USA). Graphs was drawn with GraphPad 6.0(GraphPad Software, La Jolla, CA, USA). The regulatory network was drawn base on STRING database.

### Validation of the identified SNPs

The validation cohort enrolled 204 ATB patients and 109 LTBI patients. Inclusion and exclusion criteria were the same as the prospective cohort. Patients were recruited from hospitals in Yangtze River Delta region (China) and pulmonary Hospital of Chonqing (Chongqing, China).

We genotyped the identified SNPs which were screened out by eQTLs mapping and further analyzed via multiplex SNaPshot® assay (Life Technologies, USA) in the validation cohort. Data was analyzed using SPSS 20.0 software (IBM Corp, USA) and histogram was performed to show the difference of selected SNPs between ATB and LTBI patients using GraphPad 6.0(La Jolla, USA).

## Results

We screened out 27 mRNAs for further analysis after reviewing the recent studies on biomarkers differently expressed in two TB infection status. Ninety-two individuals including 43 ATB patients and 49 LTBI patients were enrolled in the prospective cohort (Additional file 1: Figure S1). Using dcRT-MLPA, we identified 9 mRNAs with significant gene expression differences between the two groups. Furthermore, through eQTL mapping, we screened out SNPs significantly associated with the identified 9 mRNAs. And according to the function and interaction analysis, 8 SNPs were finally selected to be assessed in the validation cohort. In the validation cohort, we evaluated the identified SNPs and finally verified a new SNP rs62292160, which was shown to be associated with tuberculosis infection*.*

### Identifying differentially expressed mRNAs between ATB and LTBI cohorts

The baseline information of 43 ATB and 49 LTBI patients in the prospective cohort was assessed. The mean age of ATB patients was 44 and the mean age of LTBI patients was 49.7. Significant gender difference was found between two groups (P = 0.012). The spot count of T-SPOT.*TB* and percentage of coughing and chest tightness or pain were significantly higher in the ATB groups than that in the LTBI group, while the percentage of enrolled subjects who admitted of BCG vaccination were significantly higher in the LTBI groups (P < 0.05). The overall baseline data was shown in Table 1.

**Table 1:** Baseline information of prospective and validation cohort

	Prospective cohort		
Characteristic	ATB group (n = 43)	LTBI group (n = 49)	P value
Age (average)			
	44.0	49.7	0.057
Gender			
Male (%)	30 (69.8%)	21 (42.9%)	0.012
Female (%)	13 (30.2%)	28 (57.1%)	
T-SPOT.TB			
Panel A, spot count	88	31	0.002
Panel B, spot count	141	41	0.009
Contact with ATB			
Yes (%)	2 (4.7%)	49 (100%)	/
No (%)	41 (95.3%)	0 (0%)	
BCG vaccination history			
Yes (%)	6 (14.0%)	31 (63.3%)	0.000
No/cannot remember (%)	37 (86.0%)	18 (36.7%)	
Clinical manifestations			
Coughing with expectoration (%)	36 (83.7%)	17 (34.7%)	0.000
Chest tightness or pain (%)	4 (9.3%)	0 (0%)	0.044
Hemoptysis (%)	3 (7.0%)	0 (0%)	0.098
Night sweats (%)	1 (2.3%)	1 (2.0%)	1.000
Fever (%)	1 (2.3%)	1 (2.0%)	1.000
Inappetence (%)	0 (%)	0 (0%)	/
Lung cavity (%)	17 (39.5%)	/	/
Comorbidity			
Diabetes mellitus (%)	5 (11.6%)	1 (2.0%)	0.094
CHB (%)	6 (16.3%)	2 (4.1%)	0.140
HIV (%)	0 (0%)	0 (0%)	/
Hypertension (%)	1 (2.3%)	5 (10.2%)	0.209
Silicosis (%)	1 (2.3%)	0 (0%)	0.467

	Validation cohort		
Characteristic	LTBI (n = 104)	ATB (n = 209)	P value
Gender			
Male (%)	43	134	0.0001
Female (%)	56	66	
Age	45.17 ± 1.527	47.80 ± 1.449	0.2621
Nationality			
Han	104	209	–
District			
Chongqing	61	54	< 0.0001
Around Shanghai	43	155	

The baseline information of patients in prospective and validation cohort

*ATB* active tuberculosis, *LTBI* latent tuberculosis infection, *BCG* Bacillus Calmette-Guérin, *CHB* chronic hepatitis B, *HIV* human immunodeficiency virus

We analyzed the peripheral blood expression levels of 27 selected mRNAs using dcRT-MLPA and finally identified 9 differentially expressed (p < 0.05) mRNAs including *il7r, il4, il8, tnfrsf1b, pgm5, ccl19, il2ra, marco*, and *fpr1*. The peripheral blood gene expression levels of *il7r, pgm5, ccl19, marco*, and *fpr1* were statistically up-regulated in ATB patients (p < 0.05), whereas the expression levels of *il4, il8, tnfrsf1b*, and *il2ra* were significantly up-regulated in LTBI groups (p < 0.05). There were no significant differences in the expression levels of the rest 18 genes (Table 2 and Additional file 1: Figure S2).

**Table 2:** Analysis of differential expression of whole blood mRNA in ATB and LTBI patients by dcRT-MLPA method

mRNA	ATB	LTBI	P value	mRNA	ATB	LTBI	P value	mRNA	ATB	LTBI	P value
*Il4*	5.52	6.73	< 0.0001	*Sec14l*	2.859	3.2	0.0592	*Timp2*	8.155	8.266	0.5858
*Il7r*	9.15	8.147	< 0.0001	*ltf*	7.125	6.53	0.0761	*Casp8*	12.26	12.36	0.6134
*Il8*	9.254	9.844	< 0.0001	*Mmp9*	6.34	6.155	0.0969	*Tnfrsf1a*	12.04	12.01	0.7111
*Tnfrsf1b*	9.93	10.42	0.0001	*Ccr7*	13.02	12.93	0.1686	*Ifng*	9.85	9.82	0.717
*Pgm5*	9.11	7.982	0.0016	*Bcl2*	3.825	4.19	0.2555	*Tgfb1*	12.68	12.68	0.7831
*Ccl19*	9.28	8.435	0.0034	*Cd4*	5.815	5.71	0.3819	*Cd8a*	8.735	8.66	0.8726
*Il2ra*	7.445	7.784	0.0143	*Il6*	4.472	4.1	0.4205	*Foxp3*	11.04	10.96	0.8905
*Marco*	5.98	5.44	0.0174	*Tnfrsf10c*	10.38	10.48	0.4313	*Blr1*	6.32	6.336	0.9785
*Fpr1*	4.47	4.178	0.0206	*Fcgr1a*	5.03	5.041	0.4422	*Cxcl10*	10.41	10.37	0.9953

The 9 mRNAs in the first column showed significant differences in expression levels between the ATB and LTBI groups. Among them, *il7r, pgm5, ccl19, marco,* and *fpr1* were up-regulated in ATB patients. *Il4, il8, tnfrsf1b,* and *il2ra* were up-regulated in LTBI patients

*ATB* active tuberculosis, *LTBI* latent tuberculosis infection

We further drew the receiver operating characteristic (ROC) curve and the area under the curves (AUCs) of identified 9 genes were 0.7635, 0.7633, 0.763, 0.7253, 0.6888, 0.6753, 0.6489, 0.643 and 0.6393, respectively (*il7r, il4, il8, tnfrsf1b, pgm5, ccl19, il2ra, marco* and *fpr1*) (Additional file 1: Figure S3).

### Screening out specific SNPs associated with differentially expressed mRNAs using eQTLs analysis

**Fig. 1:**
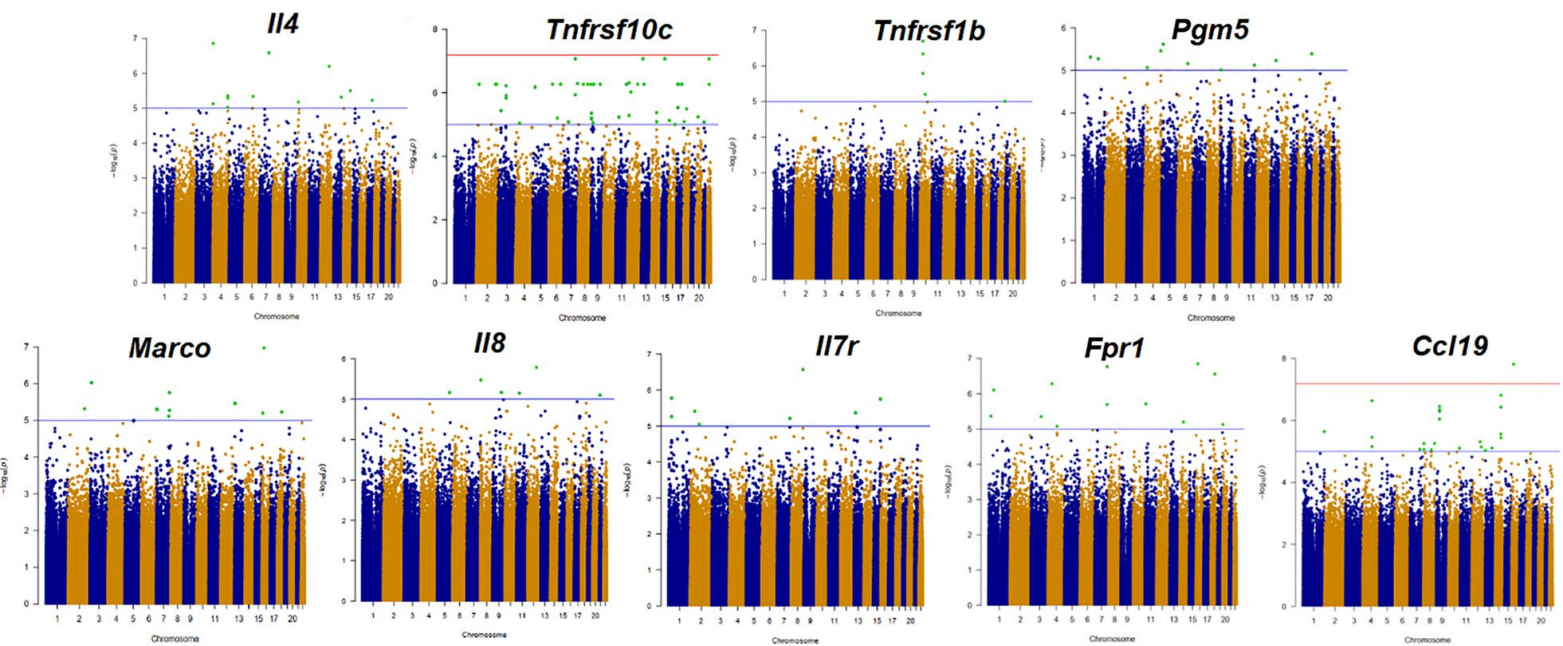
Manhattan plot. The expression levels of mRNAs were treated as quantitative traits and were associated with single nucleotide polymorphisms. The p value of green plots was less than E^−05^. The blue line represents E−05 and the red lines for E^−07^

**Fig. 2:**
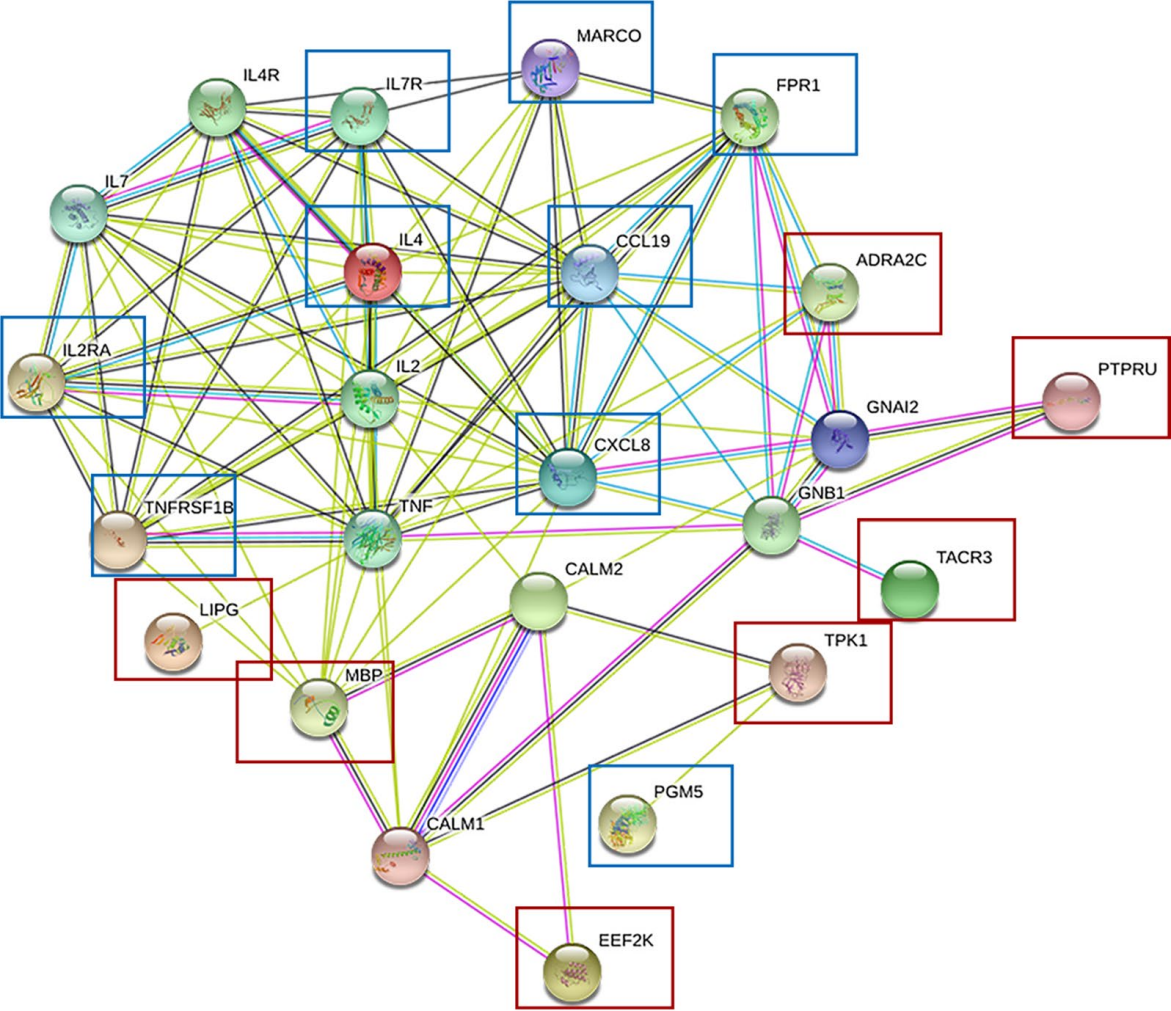
Regulatory network of significantly differentially expressed mRNAs and SNPs related genes. Regulatory network of mRNAs and SNPs related genes. The genes were surrounded by a red rectangle and the RNAs were surrounded by a blue rectangle. Seven genes and 9 mRNAs were in this network

We extracted DNA from peripheral blood mononuclear cells and obtained genome-wide SNPs information. Then we associated the SNPs with the 9 identified mRNAs. After eQTL mapping, we selected out the significant SNPs with the following strategy (Table 3). Firstly, 107 SNPs with p values less than E^−05^ were selected out (Fig. 1 and Additional file 1: Table S4). In the 107 SNPs, rs7203246, rs62292160, rs17170200, rs2189164 and rs3786247 were shown to have smallest p values (related genes were EEF2K, ADRA2C, TPK1, TACR3 and LIPG respectively). We then analyzed the function of 107 SNPs-related genes. Some were previously reported to participate in the process of human immune regulation or TB infection and these were also included for the future validation study. Among those, rs1667908-related gene was MBP and it translated protein which was found to express ubiquitously, especially in the immune system. And the protein might regulate proliferation and activation of T cells negatively [14]. Rs7203246-related gene was EEF2K, the gene coded the protein which was found to have effect in the differentiation of lung fibroblast through p38 mitogen activated protein kinases (MAPK) signal pathway [15]. Rs12712986 was related to gene TTC7A. TTC7A may have some effect in immunity and mutation of this gene was reported to be associated with inflammatory bowel disease [16]. What’s more, we analyzed the interaction relationship of SNPs-related genes and differentially expressed mRNAs previously identified (Fig. 2). Seven SNPs, rs4654400, rs7203246, rs62292160, rs2189164, rs3786247, rs1667908, and rs17170200 related genes were in the regulatory network. After comprehensive consideration of the p value, function of related genes and interaction among genes and identified mRNAs, we finally screened out 8 SNPs, rs7203246, rs62292160, rs17170200, rs2189164, rs3786247, rs12712986, rs4654400 and rs1667908 for the validation.

**Table 3:** Selected SNPs, related genes and corresponding selection strategies

Strategies	SNPs	Related Genes
The expression levels of mRNAs associated with SNPs using eQTL analysis(P < E-05)	107 SNPs (see Additional file 1: Table S4)	**-**
The 5 SNPs with smallest p values	rs7203246	EEF2K
	rs62292160	ADRA2C
	rs17170200	TPK1
	rs2189164	TACR3
	rs3786247	LIPG
Related genes were involved in the modulation of immune functions or playing a role TB	rs1667908	MBP
	rs7203246	EEF2K
	rs12712986	TTC7A
Related genes interact in a regulatory network	rs4654400	PTPRU
	rs7203246	EEF2K
	rs62292160	ADRA2C
	rs2189164	TACR3
	rs3786247	LIPG
	rs1667908	MBP
	rs17170200	TPK1

The table showed the strategy for screening the SNPs. First, 107 SNPs whose P value < E−05 were selected. In these 107 SNPs, the most differentially expressing 5 SNPs were selected, whose p value was less than E−07. 3 SNPs which played key roles in immune regulation or TB and 7 SNPs involved in the interaction network were select

### Validation of the identified SNPs

**Fig. 3:**
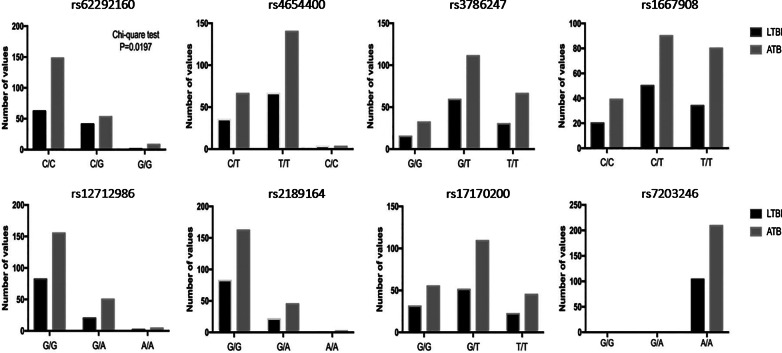
Genotypes of 8 Sigle Nucleoside polymorphisms. We validated the identified SNPs and the histogram was performed to show the variations of selected SNPs in ATB and LTBI patients. The rs62292160 whose related gene was ADRA2C had significant differences in ATB and LTBI patient

Two-hundred and nine ATB and 104 LTBI patients were enrolled in the validation cohort. There were no significant differences in the mean age in ATB and LTBI groups. All patients were Han Chinese and were recruited from multi-centers. These patients were from Yangtze River Delta region and southwest area of China (Table 1). Eight selected SNPs were analyzed and successfully, we found the C/G genotype of rs62292160 showed significantly higher percentage in the LTBI patients and CC genotype of rs62292160 showed significantly higher in ATB patients. However, no variations of the rest 7 SNPs were found in the validation cohort (Fig. 3).

## Discussions

Up to now, TB remains to be one of the top 10 causes of morbidity and mortality around the world [1]. The challenge from TB is due to many reasons, one of which is the existence of a large population infected with TB latently, and 10% of which will develop ATB during their lifetime [2]. The treatment for ATB and LTBI were different and high treatment coverage of LTBI is equally important to that of ATB. It can help to reduce the incidence of ATB and the burden of TB eventually. Currently, tuberculin skin test (TST) and interferon-gamma release assays (IGRAs) cannot discriminate between ATB and LTBI groups. And it is still unclear why some LTBI patients will develop ATB but others will not. The WHO guideline described at-risk groups who might progress to ATB, including patients with HIV, diabetes, chronic renal failure and so on [17]. Our study explored the characteristics of susceptible population at the molecular level and tried to explain the molecular mechanisms of susceptible population to TB in Chinese Han population.

Thye et al. have already identified rs4331426 and rs2057178 in TB patients by GWAS [9, 10]. The gene mutations of different ethnics were different and the same gene mutation might have opposite effects in different ethnics [18]. In recent years, researchers successively found the SNPs specific to Chinese Han population including rs1946518, rs5744247, rs1800796 and so on [18–21]. Although using GAWS or SNaPshot assay could associate gene variations with disease phenotypes, that could not explain the mechanisms underlying actions of SNPs. Chen et al. tried to explain the effects of SNPs in a Chinese cohort by correlating gene expression levels with previously identified rs4331426 and rs2057178, and finally screened out rs2057178 which might influence the expression levels of MAFB and SOCS2 in Chinese Han population [22]. Recently, researchers used eQTL mapping to identify the relationship between gene expression levels and genotypic variations in several diseases including chronic kidney disease, tumor and insomnia. And it helped to find plenty of significant SNPs [23–25]. In our study, we used eQTL mapping to analyze the association between SNPs, mRNA from peripheral blood and tuberculosis infection phenotypes in Chinese Han population.

In the prospective cohort, we selected 27 mRNAs after reviewing the recent studies on biomarkers which can discriminate between ATB and LTBI. The expression levels of these mRNAs were mainly analyzed in groups from Europe, Africa and America, but our study further tested the expression levels of theses mRNAs in Chinese Han population in different status of TB infection. Finally, we identified 9 mRNAs differentially expressed in ATB patients and LTBI patients, including *il7r, il4, il8, tnfrsf1b, pgm5, ccl19, il2ra, marco* and *fpr1*.

All these mRNAs have been found to play key roles in human immune responses or the progressing to ATB. Human *il4* was the archetypal T-helper-2 (Th2) cytokine, serving as the critical element in the induction of Th2 response and blocked some of the effects of *il2* [26]. *il4* could subvert mycobacterial containment in human macrophages, likely via perturbations in Treg and Th1-linked pathways [27]. As for *il7r*, both Jenum et al. and Mihret et al. have found the different gene expression level of *il7r* in TB patients [28, 29]. Jenum et al. found that the *tnfrsf1b* separated ATB from LTBI patients in children [30]. *Tnfr2* was an important TNF-α receptor and it was crucial to control pleural tuberculosis [31, 32]. *Pgm5* made sense in the formation of carbohydrates and researchers have already found it expressed differentially in several diseases [33]. *Ccl19* was involved in B cell-related pathways and played key role in immune response [34]. *Marco* was essential in the inflammatory responses to bacterial pathogens and several SNPs have been proved to be associated with susceptibility to infectious diseases [35]. And *Fpr1* was detected to be important in neutrophils function-related pathways [36].

These selected mRNAs were previously proved to be critical elements in the development of infectious diseases including TB, and our cohort included a larger sample size of cases to further validate these mRNAs in Han population. Therefore, using these gene expression levels for further eQTLs mapping was more likely to find significant and meaningful genetic variations.

By the eQTL mapping analysis, we found out 8 SNPs in the prospective cohort. In the final validation step, we successfully identified a new SNP rs62292160, which showed a significant difference in ATB and LTBI patients in Chinese Han population. and that had not been reported the association with TB previously in any ethnics.

Rs62292160 was located at 4q12 and 62 Kb away from ADRA2C, which was totally a new locus. And the variation of rs62292160 was significantly associated with the higher expression level of IL4 in LTBI patients. The rs62292160 related to gene ADRA2C encodes adrenergic receptor alpha-2C (α2C-AR), which is one of the three subtypes of the alpha-2-adrenergic receptor(α2-AR). The protein α2-AR is one of the members of the G protein-coupled receptor superfamily and it has the general characteristics of G-protein coupled receptors. It can couple with G proteins of the Go/Gi-type, and inhibit the function of adenylate cyclase enzyme and the voltage-gated calcium channels. Furthermore, it plays a role in the activation of potassium channels and could also stimulate the mitogen-activated protein kinase (MAPK) [37]. The previous study suggested the decrease of alpha-adrenergic receptors in human lung tissue when patients suffered from destructive tuberculosis [38]. This suggested that the α2-ARs might be associated with TB. Bao et al. found that the α2-ARs participated in the down-regulation of cytokine production including IFN-γ and IL4 in the experiment using T lymphocytes from rats [39]. The cytokines played an essential role in combating *Mtb*. After the host is infected, *Mtb* interacts with the host to induce changes in cytokines release such as TNF-α, IFN-γ, IL8 and IL4 by affecting JAK/STAT, MAKP, calcium-dependent signaling pathways and so on [40–42]. The rs62292160-related gene ADRA2C might influence the expression of these cytokines through MAKP or calcium-dependent signaling pathways. Up to now, most studies focused on the adrenergic receptor beta(β2-AR) in lung tissue, more studies were needed to explore the function of α2-ARs in TB.

To our knowledge, this is the first application of dc-MLPA combined with eqtl mapping to analyze gene susceptibility of latent and active tuberculosis infection status. Using the technique, we have successfully found rs62292160 in the Chinese Han population, which might effect human immune responses in TB infection which leads to different infection status. On one hand, our result provides a new direction for further study of the mechanism of TB infection; and more importantly, our study raises the possible concept that receiving preventive anti-tuberculosis treatment might be more necessary for some LTBI patients with certain susceptible genes. However, such concept would still require a large amount of further clinical research and verification.

## Conclusions

In this study, we validated the differential expression levels of 9 mRNAs in latent and active TB patients in the Chinese Han population. and we identified rs62292160, which might affect human immune responses in TB infection through regulating the expression of IL4. Not only did we provide a new variation locus for further exploration of mechanisms in TB infection, but also it might be helpful for the clinical researches. Because it holds the possibility to identify susceptible population who may progress to ATB, and guide preventive treatments for latent tuberculosis in the future.

## Supplementary Information

**Additional file 1: Table S1.** The list of selected mRNAs that were reported to distinguish between ATB and LTBI in previous studies** Table S2** The probes of 27 selected mRNAs for dcRT-MLPA.** Table S3 **The reaction steps in dcRT-MLPA.** Table S4 **The location and symbol of selected 107 SNPs with p value less than E^-05^. **Figure S1.** Flow chart. **Figure S2.** 9 mRNAs with statistically differential expression in ATB and LTBI patients. **Figure S3**. The receiver operating characteristic curve (ROC) of the selected mRNA in distinguishing ATB and LTBI patients.

## Data Availability

The datasets used and/or analysed during the current study are available from the corresponding author on reasonable request.

## Abbreviations

TB: Tuberculosis
WHO: World Health Organization
Mtb: Mycobacterium tuberculosis
LTBI: Latent tuberculosis infection
ATB: Active tuberculosis
SNP: Single nucleotide polymorphism
GWAS: Genome-wide association study
eQTL: Expression quantitative trait loci
HIV: Human immunodeficiency virus
AUCs: Area under the curves
MAPK: Mitogen activated protein kinases
TST: Tuberculin skin test
Th2: T-helper-2
α2C-AR: Adrenergic receptor alpha-2C
α2-AR: Alpha-2-adrenergic receptor
MAPK: Mitogen-activated protein kinase
