# Supplementary material for: Gene expression pattern analysis using dual-color RT-MLPA and integrative genome-wide association studies of eQTL for tuberculosis suscepitibility
Source: Respir Res. 2021 Jan 20;22:23. doi: 10.1186/s12931-020-01612-9 (PMC7816316; doi:10.1186/s12931-020-01612-9)

**Additional Table S1** The list of selected mRNAs that were reported to distinguish between ATB and LTBI in previous studies.

| **Target mRNA** | **References** |
| --- | --- |
| FCGR1A, IL7R, CASP8, MMP9, TNFRSF1A, TNFRSF1B,CCR7, IFNG, TGFB1, CCL19, IL2RA, TNFRSF10C, BLR1, PGM5, FCGR1A, CXCL10, MARCO, | Maertzdorf J, Ota M, Repsilber D, Mollenkopf HJ, Weiner J, Hill PC, & Kaufmann SHE (2011) PLOS ONE 6, e26938. |
| FOXP3,IL8,IL6 | Wu B, Huang C, Kato-Maeda M, Hopewell PC, Daley CL, Krensky AM, & Clayberger C (2007) , pp. 3688-3694. Vascotto F, Le Roux D, Lankar D, Faure-Andre G, Vargas P, Guermonprez P, & Lennon-Dumenil A (2007) CURR OPIN IMMUNOL 19, 93-98. |
| IL7R | Rane L, Rahman S, Magalhaes I, Ahmed R, Spangberg M, Kondova I, Verreck F, Andersson J, Brighenti S, & Maeurer MJ (2011) GENES IMMUN 12, 513-522.  Sudo T, Nishikawa S, Ohno N, Akiyama N, Tamakoshi M, Yoshida H, & Nishikawa S (1993) P NATL ACAD SCI USA 90, 9125-9129. |
| IL4,IL7R | Armitage RJ, Ziegler SF, Beckmann MP, Idzerda RL, Park LS, & Fanslow WC (1991) ADV EXP MED BIOL 292, 121-130. |
| CD4,CCLX10 | Ahlers JD & Belyakov IM (2010) BLOOD 115, 1678-1689. |
| IL7R | Jiang Q, Li WQ, Aiello FB, Mazzucchelli R, Asefa B, Khaled AR, & Durum SK (2005) CYTOKINE GROWTH F R 16, 513-533. |
| LTF | Jacobsen M, Repsilber D, Gutschmidt A, Neher A, Feldmann K, Mollenkopf HJ, Ziegler A, & Kaufmann SHE (2007) Journal of molecular medicine (Berlin, Germany) 85, 613-621. |
| BCL2 | Mihret A, Loxton AG, Bekele Y, Kaufmann SHE, Kidd M, Haks MC, Ottenhoff THM, Aseffa A, Howe R, & Walzl G (2014) *BMC INFECT DIS* **14,** 257 |
| SEC14L1, TIMP2 | Jenum S, Dhanasekaran S, Lodha R, Mukherjee A, Kumar Saini D, Singh S, Singh V, Medigeshi G, Haks MC, & Ottenhoff THM*, et al.* (2016) *SCI REP-UK* **6** |
| BCL2, IL7R, FCGR1A, MARCO, MMP9, CCL19, LTF, TGFB1, FOXP3 | Mihret, A., et al., Combination of gene expression patterns in whole blood discriminate betweentuberculosis infection states. BMC infectious diseases, 2014. 14: p. 257. |
| BLR1, CD8A, IL7R, FPR1, IL4, TIMP2, TNFRSF1B, FCGR1A, MMP9, SEC14L1 | Jenum, S., et al., Approaching a diagnostic point-of-care test for pediatric tuberculosis throughevaluation of immune biomarkers across the clinical disease spectrum. Scientific reports, 2016. 6: p. 18520. |

**Additional Table S2** The probes of 27 selected mRNAs for dcRT-MLPA

| **Gene** | **LPO** | **RPO** |
| --- | --- | --- |
| IL4 | GGCCGCGGGAATTCGATTCAGACATCTTTGGCTGCCTCCAAG | AACACAACTGAGAAGGAAACCTTCTGCAGGGCTGCGACTGCACTAGTGAATTCGCGGC |
| IL7R | GGCCGCGGGAATTCGATTCCCCGATCATAAGAAGACTCTGGAACATCTTTGTAAGAAACCAAGAAAA | AATTTAAATGTGAGTTTCAATCCTGAAAGTTTCCTGGACTGCCAGATTCATCACTAGTCAATTCGCGGC |
| IL8 | GGGTTCCCTAAGGGTTGGAGCCTTCCTGATTTCTGCAGCTCTG | TGTGAAGGTGCAGTTTTGCCAAGGAGTGCTAAAGAATCTAGATTGGATCTTGCTGGCAC |
| TNFRSF1B | GGGTTCCCTAAGGGTTGGAGCCAGCACCGGGAGCTCAGATTCTTCC | CCGGCCTGGTGGCCATGGGACCCAGGTCAATGTCACGTGCATCGTGAACGTCTGTAGACGCTCTGACCACACTCTAGATTGGATCTTGCTGGCAC |
| PGM5 | GGGTTCCCTAAGGGTTGGAAGCCACTGCCCTGGAGACCAGGGGGAGATTTGGAGTGAAGTTTAA | TGTTGCCAATGGAGGTCCTGCACCCGATTGTGTCTAGATTGGATCTTGCTGGCAC |
| CCL19 | GGGTTCCCTAAGGGTTGGAGGGTAGAACGCATCATCCAGAGACTGCAGAGGACCTCAGCCAAGAT | GAAGAGCCGCAGAGTTAACCTATGACCGTTCTAGATTGGATCTTGCTGGCAC |
| IL2RA | GGCCGCGGGAATTCGATTGTCCATATTTACAACAGAGTACCAGGTAGC | AGTGGCCGGCTGTGTTTTCCTGCTGATCCACTAGTGAATTCGCGGC |
| MARCO | GGCCGCGGGAATTCGATTGGACAAGCTGGCCAGAAGGGAGACCAGGGAGTGAAAGGAT | CTTCTGGGGAGCAAGGAGTAAAGGGAGAAACACTAGTGAATTCGCGGC |
| FPR1 | GGGTTCCCTAAGGGTTGGAACTAGAACTACCCAGAGCAAGACCACAGCTGGTGAACAGTCCA | GGAGCAGACAAGATGGAGACAAATTCCTCTTCTAGATTGGATCTTGCTGGCAC |
| SEC14L1 | GGCCGCGGGAATTCGATTCCAGGGTATTTCCTGTGCTCTGGACGCTGGTT | AGTCCGTTCATTGATGACAACACCAGAAGGAAGTCACTAGTGAATTCGCGGC |
| LTF | GGCCGCGGGAATTCGATTGACATGAAACTTGTCTTCCTCGTCC | CGGACTGTGTCTGGCTGGCCGTAGGAGGAGTGTTCAGTCACTAGTGAATTCGCGGC |
| MMP9 | GGCCGCGGGAATTCGATTCCTGAGAACCAATCTCACCGACAGGCAGCTGGCAGAGGAA | TACCTGTACCGCTATGGTTACACTCGGGTGGCAGAGATCACTAGTGAATTCGCGGC |
| CCR7 | GGGTTCCCTAAGGGTTGGATGGTTTTACCGCCCAGAGAGCGTCATGGACCTGG | GGAAACCAATGAAAAGCGTGCTGGTGGTGGCTCTCCTTGTCATTTTCCAGGTATCTAGATTGGATCTTGCTGGCAC |
| BCL2 | GGGTTCCCTAAGGGTTGGACTTGACAGAGGATCATGCTGTACTTAAAAAATACAACATCA | CAGAGGAAGTAGACTGATATTAACAATACTTACTAATAATAACCTGCCTCATGAAATAAAGATCTAGATTGGATCTTGCTGGCAC |
| CD4 | GGCCGCGGGAATTCGATTGGCACTGGCTTCTGGTGCTGCAACTG | GCGCTCCTCCCAGCAGCCACTAAGGGAACACTAGTGAATTCGCGGC |
| IL6 | GGGTTCCCTAAGGGTTGGAATGCTTCCAATCTGGATTCAATGAG | GAGACTTGCCTGGTGAAAATCATCACTGGTCTTTTGGATTTTGTCTAGATTGGATCTTGCTGGCAC |
| TNFRSF10C | GGGTTCCCTAAGGGTTGGAACATACTGTAGCCTGTAACCCGTGCACAGAGGGTGTGGATTACACCAACGCTTCCAAC | AATGTTCCTTCTTCTTCCCATGTACAGTTTGTAAATCAGATCAAAAACATCTAGATTGGATCTTGCTGGCAC |
| FCGR1A | GGCCGCGGGAATTCGATTGACAACATGTGGTTCTTGACAACTCTGCTCCTTTGGGTTCCAGTTGATGGGCAA | GTGGACACCACAAAGGCAGTGATCACTTTGCAGCCTCCATGGGTCACACTAGTGAATTCGCGGC |
| TIMP2 | GGGTTCCCTAAGGGTTGGAAGGCGTTTTGCAATGCAGATGTAGTG | ATCAGGGCCAAAGCGGTCAGTGAGAAGGAATCTAGATTGGATCTTGCTGGCAC |
| CASP8 | GGCCGCGGGAATTCGATTTTCTTTTGCAAGAGGAAATCTCCAAATGCAAACTGGATGATGACATGAAC | CTGCTGGATATTTTCATAGAGATGGAGAAGAGGGTCATCCTGGGTGAAGGAAAGTTGGCACTAGTGAATTCGCGGC |
| TNFRSF1A | GGCCGCGGGAATTCGATTCCTCTTCATTGGTTTAATGTATCGCTACCAACGGTGGAAGTCCAAGCTCTACTCCATTG | TTTGTGGGAAATCGACACCTGAAAAAGAGGGGGAGCTGGAAGGAACACTAGTGAATTCGCGGC |
| IFNG | GGGTTCCCTAAGGGTTGGAGATGACTTCGAAAAGCTGACTAATTATTCG | GTAACTGACTTGAATGTCCAACGCAAAGCAATACTCTAGATTGGATCTTGCTGGCAC |
| TGFB1 | GGCCGCGGGAATTCGATTCTTTTGATGTCACCCGGAGTTGTGCGGCAGTTGTTGAGCCGTAGGAGGGGAAAT | TGAGGGCTTTCGCCTTAGCGCCCACTGCTCCTGTGACACACTAGTGAATTCGCGGC |
| CD8A | GGCCGCGGGAATTCGATTTCCATCATGTACTTCAGCCACTTCGTGCCGGTCTTCCTGC | CAGCGAAGCCCACCACGACGCCAGCGCCGCGACCACCAACACCGGCCACTAGTGAATTCGCGGC |
| FOXP3 | GGGTTCCCTAAGGGTTGGACACCTGGCTGGGAAAATGGCACTGACCAAGGCTTCATCTGTG | GCATCATCCGACAAGGGCTCCTGCTGCATCGTAGCTGCTGGTCTAGATTGGATCTTGCTGGCAC |
| BLR1 | GGCCGCGGGAATTCGATTCGAGAACCTGGAGGACCTGT | TCTGGGAACTGGACAGATTGGACAACTATAACGACACCTCCCCACTAGTGAATTCGCGGC |
| CXCL10 | GGGTTCCCTAAGGGTTGGACTTTCTGACTCTAAGTGGCATTCA | AGGAGTACCTCTCTCTAGAACTGTACGCTCTAGATTGGATCTTGCTGGCAC |
| B2M | GGGTTCCCTAAGGGTTGGAGGCCTGGAGGCTATCCAGCTTA | CTCCAAAGATTCAGGTTTACTCACGTTCTAGATTGGATCTTGCTGGCAC |

LPO: left probe, RPO: right probe. B2M was the inner referenc

**Additional Table S3** The reaction steps in dcRT-MLPA

**Reaction system 1**

| **Reagent** | **Volume** |  |
| --- | --- | --- |
| SALSA MLPA buffer | 1.5ul |  |
| MLPA half-probemix(4nM) | 1.5ul |  |
| Specific reverse transcription cDNA | 6ul |  |
| Total | 9ul |  |

**Reaction step 1**

| **Step** | **Time** | **Temperature** |
| --- | --- | --- |
| Denaturation | 60s | 95℃ |
| Hybridization | 20h | 60℃ |
| Pause | ∞ | 54℃ |

**Reaction system 2**

| **Reagent** | **Volume** |  |
| --- | --- | --- |
| Reaction system 1 | 9ul |  |
| SALSA Ligase Buffer A | 3ul |  |
| SALSA Ligase Buffer | 3ul |  |
| SALSA Ligase-65 enzyme | 1ul |  |
| dH2O | 25ul |  |

**Reaction step 2**

| **Step** | **Time** | **Temperature** |
| --- | --- | --- |
| Ligation | 30min | 54℃ |
| Inactivation of enzyme | 5min | 98℃ |
| Pause | ∞ | 20℃ |

**Reaction system 3**

| **Reagent** | **Volume** |  |
| --- | --- | --- |
| Reaction system 2 | 5ul |  |
| MLPA+MAPH primer | 1+1ul |  |
| TaKaRa PCR mix | 10ul |  |
| dH2O | 3ul |  |
| Total | 20ul |  |

**Reaction step 3**

|  | **Time** | **Temperature** |
| --- | --- | --- |
|  | 5min | 95℃ |
| 33 cycles | 30s | 95℃ |
|  | 30s | 60℃ |
|  | 30s | 72℃ |
|  | 10min | 72℃ |
|  | ∞ | 4℃ |

**Additional Table S4** The location and symbol of selected 107 SNPs with p value less than E^-05^

| dbSNP_ID | CHR | BP | P | -log10 | SYMBOL |  |
| --- | --- | --- | --- | --- | --- | --- |
| rs7203246 | 16 | 22203005 | 1.54E-08 | 7.81247928 | LOC101930123;EEF2K/NA |  |
| rs7203246 | 16 | 22203005 | 1.05E-07 | 6.98088371 | LOC101930123;EEF2K/NA |  |
| rs62292160 | 4 | 3832662 | 1.39E-07 | 6.85855023 | ADRA2C |  |
| rs7203246 | 16 | 22203005 | 1.41E-07 | 6.84955059 | LOC101930123;EEF2K/NA |  |
| rs1767418 | 14 | 85709382 | 1.53E-07 | 6.81530857 | NA |  |
| rs17170200 | 7 | 144287580 | 1.70E-07 | 6.76878535 | TPK1/NA |  |
| rs17496856 | 10 | 35208068 | 2.01E-07 | 6.69615623 | NA |  |
| rs2189223 | 4 | 104695124 | 2.31E-07 | 6.63695241 | TACR3 |  |
| rs57549814 | 4 | 104695431 | 2.31E-07 | 6.63695241 | TACR3 |  |
| rs62474018 | 7 | 112049666 | 2.57E-07 | 6.58939146 | NA |  |
| rs1514626 | 8 | 132765741 | 2.72E-07 | 6.56511188 | EFR3A |  |
| rs9958734 | 18 | 47118398 | 2.74E-07 | 6.56177419 | LIPG |  |
| rs3786247 | 18 | 47118923 | 2.74E-07 | 6.56177419 | LIPG |  |
| rs4878191 | 9 | 38290694 | 3.54E-07 | 6.45050629 | NA |  |
| rs1667505 | 14 | 85706196 | 3.73E-07 | 6.42805836 | NA |  |
| rs11846871 | 14 | 85715670 | 3.73E-07 | 6.42805836 | NA |  |
| rs1449414 | 14 | 85721807 | 3.73E-07 | 6.42805836 | NA |  |
| rs2840227 | 9 | 38297774 | 4.57E-07 | 6.34046409 | NA |  |
| rs7080674 | 10 | 35244844 | 0.00000046 | 6.33724217 | NA |  |
| rs763935 | 9 | 38298279 | 5.16E-07 | 6.28751866 | NA |  |
| rs7674353 | 4 | 47533838 | 5.19E-07 | 6.28474897 | ATP10D/NA |  |
| rs4357750 | 12 | 101618037 | 6.33E-07 | 6.19880217 | NA |  |
| rs117276055 | 1 | 60818021 | 7.82E-07 | 6.10690433 | NA |  |
| rs1442977 | 9 | 38299419 | 9.07E-07 | 6.0424406 | NA |  |
| rs13091225 | 3 | 14697590 | 9.34E-07 | 6.02988568 | CCDC174 |  |
| rs73022117 | 3 | 14706770 | 9.34E-07 | 6.02988568 | C3orf20/CCDC174 |  |
| rs3773490 | 3 | 14709935 | 9.34E-07 | 6.02988568 | C3orf20/CCDC174 |  |
| rs2975 | 3 | 14716371 | 9.34E-07 | 6.02988568 | C3orf20/CCDC174 |  |
| rs7633080 | 3 | 14719946 | 9.34E-07 | 6.02988568 | C3orf20/CCDC174 |  |
| rs76413398 | 10 | 35193277 | 1.63E-06 | 5.78727985 | NA |  |
| rs4357750 | 12 | 101618037 | 1.66E-06 | 5.78041547 | NA |  |
| rs12117137 | 1 | 34205134 | 1.68E-06 | 5.77469072 | CSMD2/NA |  |
| rs17170200 | 7 | 144287580 | 1.73E-06 | 5.76245626 | TPK1/NA |  |
| rs57170842 | 15 | 98235463 | 1.79E-06 | 5.74763249 | NA |  |
| rs11822766 | 11 | 11473089 | 1.93E-06 | 5.71399288 | GALNT18 |  |
| rs10231525 | 7 | 144308410 | 2.01E-06 | 5.69594053 | TPK1/NA |  |
| rs3124102 | 1 | 246697683 | 2.30E-06 | 5.63827216 | NA |  |
| rs2962211 | 5 | 18745377 | 2.43E-06 | 5.61475132 | NA |  |
| rs2888433 | 14 | 85716792 | 2.74E-06 | 5.56256656 | NA |  |
| rs3809404 | 14 | 101356010 | 3.15E-06 | 5.50141379 | NA |  |
| rs17069257 | 8 | 4043515 | 3.38E-06 | 5.47095483 | NA |  |
| rs6996352 | 8 | 4045835 | 3.38E-06 | 5.47095483 | NA |  |
| rs17076551 | 13 | 22869583 | 3.42E-06 | 5.46546624 | NA |  |
| rs9580346 | 13 | 22875481 | 3.42E-06 | 5.46546624 | NA |  |
| rs6490719 | 13 | 22876310 | 3.42E-06 | 5.46546624 | NA |  |
| rs6490723 | 13 | 22883992 | 3.42E-06 | 5.46546624 | NA |  |
| rs6834709 | 4 | 182295274 | 3.48E-06 | 5.45879531 | NA |  |
| rs2189164 | 4 | 104725088 | 3.49E-06 | 5.45729903 | TACR3 |  |
| rs12100975 | 14 | 87124246 | 3.61E-06 | 5.44309473 | FLRT2 |  |
| rs12712986 | 2 | 47273668 | 0.00000388 | 5.41116827 | CALM2/TTC7A/NA |  |
| rs7225173 | 17 | 65001285 | 4.07E-06 | 5.39008559 | CACNG4/NA |  |
| rs1924187 | 13 | 38749184 | 4.31E-06 | 5.36562351 | LINC00571 |  |
| rs4654400 | 1 | 29584271 | 4.31E-06 | 5.36522054 | PTPRU/NA |  |
| rs2612691 | 4 | 175411381 | 4.40E-06 | 5.35644863 | HPGD |  |
| rs78793415 | 3 | 117526062 | 0.00000442 | 5.35457773 | NA |  |
| rs76303424 | 6 | 99171103 | 4.56E-06 | 5.34065436 | NA |  |
| rs16867269 | 2 | 181094327 | 4.78E-06 | 5.32048126 | CWC22 |  |
| rs277846 | 13 | 109747382 | 4.82E-06 | 5.3165927 | MYO16/NA |  |
| rs55914748 | 1 | 82884721 | 4.85E-06 | 5.31416873 | NA |  |
| rs10859690 | 12 | 94638309 | 4.89E-06 | 5.31060234 | PLXNC1 |  |
| rs4719457 | 7 | 2230225 | 4.91E-06 | 5.30918394 | MAD1L1 |  |
| rs2060163 | 7 | 7173681 | 5.04E-06 | 5.29791428 | C1GALT1 |  |
| rs1426946 | 4 | 175390534 | 5.11E-06 | 5.29132421 | HPGD |  |
| rs10231525 | 7 | 144308410 | 5.28E-06 | 5.27711939 | TPK1/NA |  |
| rs58049435 | 1 | 170960708 | 0.00000535 | 5.27164622 | MROH9 |  |
| rs481776 | 1 | 34205859 | 5.49E-06 | 5.2601904 | CSMD2/NA |  |
| rs4870904 | 8 | 125578501 | 5.53E-06 | 5.25758912 | MTSS1/NDUFB9 |  |
| rs17063168 | 8 | 3677594 | 5.58E-06 | 5.25305459 | CSMD1 |  |
| rs9958734 | 18 | 47118398 | 5.84E-06 | 5.23373591 | LIPG |  |
| rs3786247 | 18 | 47118923 | 5.84E-06 | 5.23373591 | LIPG |  |
| rs1930333 | 13 | 81066539 | 5.85E-06 | 5.23291838 | NA |  |
| rs792365 | 17 | 55453995 | 5.90E-06 | 5.22936887 | MSI2/NA |  |
| rs17170200 | 7 | 144287580 | 6.18E-06 | 5.2090818 | TPK1/NA |  |
| rs1395583 | 16 | 7594652 | 6.28E-06 | 5.20197121 | RBFOX1/NA |  |
| rs7151850 | 14 | 63164160 | 6.32E-06 | 5.19948912 | KCNH5 |  |
| rs16913479 | 10 | 61113279 | 0.00000633 | 5.19859629 | FAM13C |  |
| rs11817272 | 10 | 11297711 | 6.65E-06 | 5.1769172 | CELF2/NA |  |
| rs735111 | 9 | 100895443 | 6.83E-06 | 5.16564289 | CORO2A/TRIM14 |  |
| rs10985166 | 9 | 100896048 | 6.83E-06 | 5.16564289 | CORO2A/TRIM14 |  |
| rs17103640 | 5 | 145081944 | 6.87E-06 | 5.16291685 | PRELID2 |  |
| rs9387075 | 6 | 112718049 | 6.93E-06 | 5.15945481 | NA |  |
| rs36096554 | 4 | 104757193 | 6.96E-06 | 5.15745316 | TACR3 |  |
| rs17034148 | 4 | 104778948 | 6.96E-06 | 5.15745316 | TACR3 |  |
| rs189591476 | 11 | 35349822 | 7.16E-06 | 5.14496568 | SLC1A2/NA |  |
| rs5001158 | 12 | 107872729 | 7.24E-06 | 5.14032142 | BTBD11/NA |  |
| rs636293 | 20 | 4328509 | 7.42E-06 | 5.12953757 | NA |  |
| rs28605619 | 4 | 3804286 | 7.43E-06 | 5.12895274 | ADRA2C |  |
| rs652243 | 11 | 107470916 | 0.00000751 | 5.12436006 | ELMOD1;LOC643923 |  |
| rs10435421 | 7 | 136339058 | 0.00000764 | 5.11690664 | NA |  |
| rs1328054 | 13 | 97054658 | 7.79E-06 | 5.10846254 | HS6ST3/NA |  |
| rs1025512 | 10 | 127237449 | 7.88E-06 | 5.10341867 | TEX36-AS1 |  |
| rs12766664 | 10 | 127237755 | 7.88E-06 | 5.10341867 | TEX36-AS1 |  |
| rs4962471 | 10 | 127239628 | 7.88E-06 | 5.10341867 | TEX36-AS1 |  |
| rs117488539 | 21 | 21631492 | 7.99E-06 | 5.09739887 | NA |  |
| rs2189164 | 4 | 104725088 | 8.28E-06 | 5.08175991 | TACR3 |  |
| rs79121908 | 7 | 114961472 | 8.55E-06 | 5.06813549 | LINC01392 |  |
| rs17735010 | 4 | 29743168 | 8.59E-06 | 5.06580465 | NA |  |
| rs9694077 | 8 | 3678385 | 8.63E-06 | 5.0641402 | CSMD1 |  |
| rs6469158 | 8 | 89011448 | 8.85E-06 | 5.05290954 | NA |  |
| rs1517682 | 2 | 99450831 | 8.93E-06 | 5.04909991 | KIAA1211L/NA |  |
| rs17076551 | 13 | 22869583 | 9.29E-06 | 5.03179733 | NA |  |
| rs9580346 | 13 | 22875481 | 9.29E-06 | 5.03179733 | NA |  |
| rs6490719 | 13 | 22876310 | 9.29E-06 | 5.03179733 | NA |  |
| rs6490723 | 13 | 22883992 | 9.29E-06 | 5.03179733 | NA |  |
| rs11133038 | 4 | 175394662 | 9.32E-06 | 5.03067729 | HPGD |  |
| rs10759064 | 9 | 9405003 | 0.00000977 | 5.01010544 | PTPRD/NA |  |
| rs1667908 | 18 | 74833456 | 9.87E-06 | 5.00577086 | MBP/NA |  |

**Additional figure S1.** Flow chart

**Recruit Prospective cohort**

43 ATB and 49 LTBI patients

**Recruit Validation cohort**

209 ATB and 104 LTBI patients

Assessing the expression level of 27 selected mRNAs between groups

dcRT-MLPA

Screening for SNPs that was associated with significant gene expression variation

eQTL

Selecting the most relevant SNPs

Selection strategy

Inclusion and exclusion criteria

Clinical data

Baseline information

Validate the previously selected SNPs between groups

Obtaining genetic loci that was associated with human immune status after TB infection

ATB: active tuberculosis; LTBI: latent tuberculosis infection; dcRT-MLPA: dual color reverse transcription multiplex ligation dependent probe amplification assay; eQTL: expression quantitative trait loci; SNP: single nucleotide polymorphism.

**Additional Figure S2a.** 9 mRNAs with statistically differential expression in ATB and LTBI patients


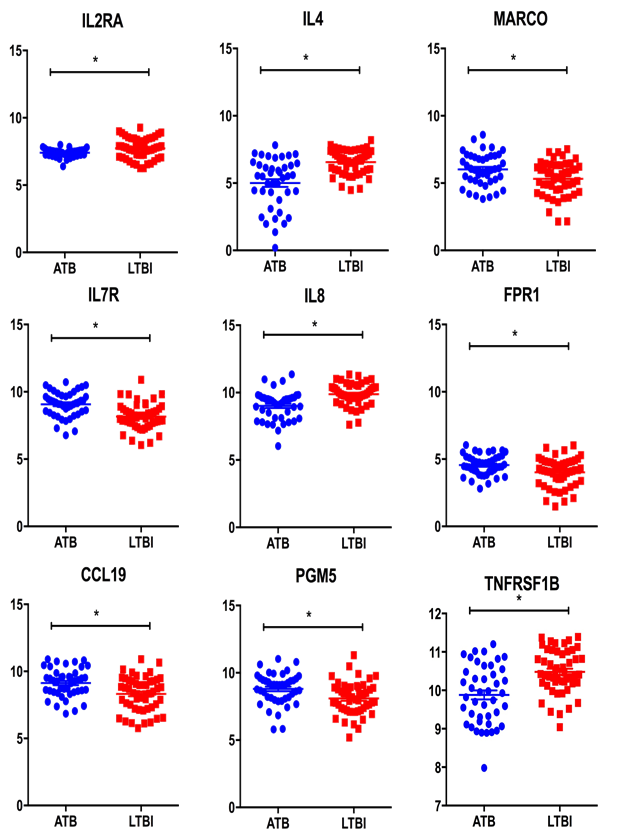


*P<0.05

**Additional Figure S2b.** 18 mRNAs without statistically differential expression in ATB and LTBI patients


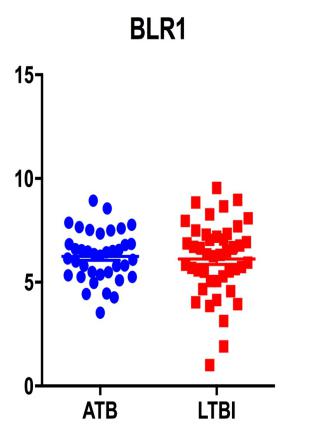

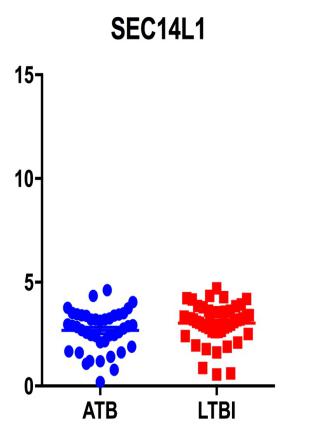

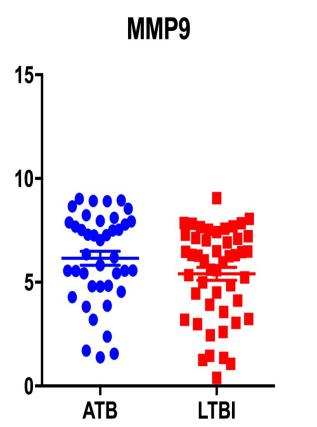

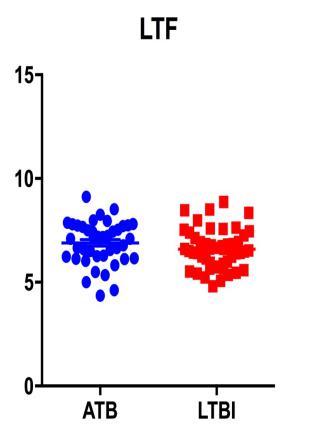

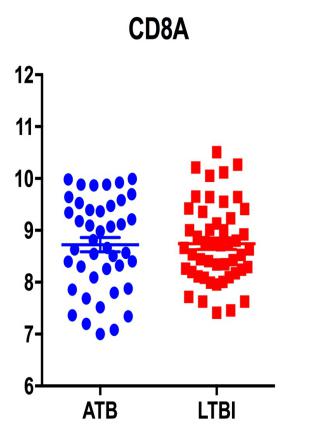

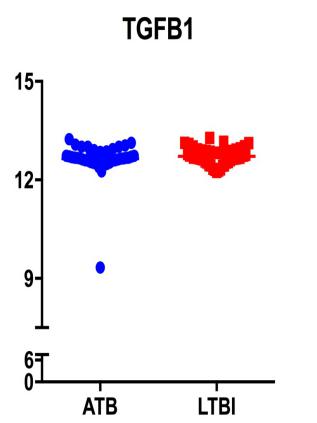

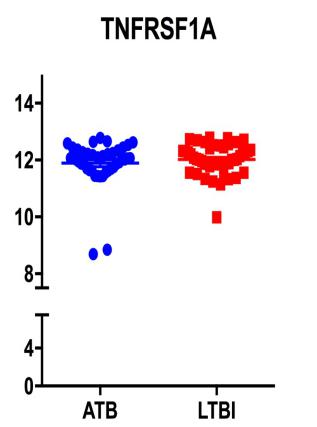

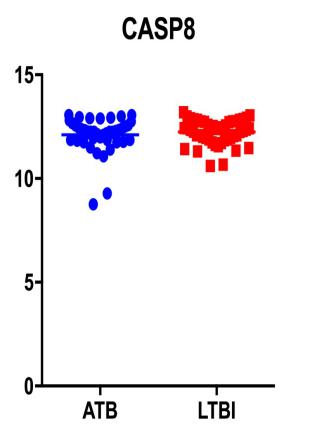

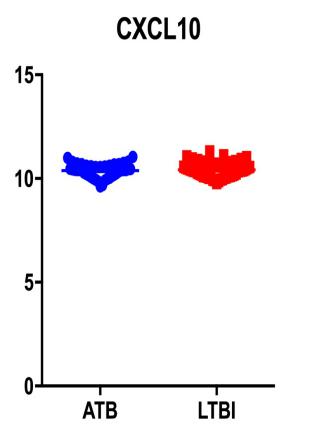

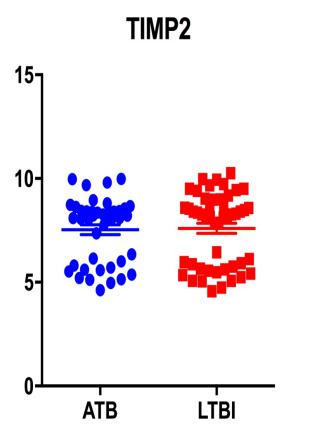

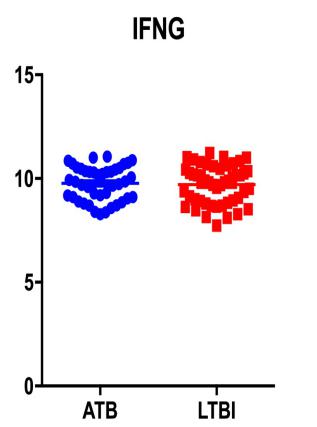

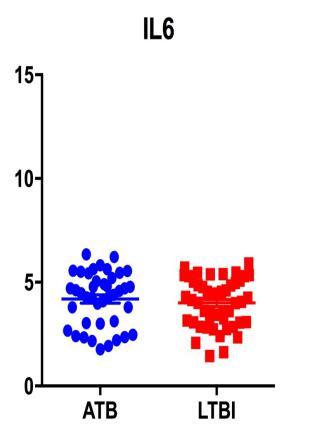

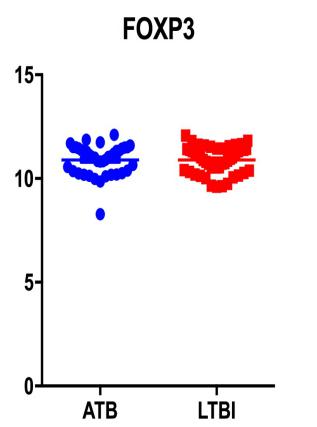

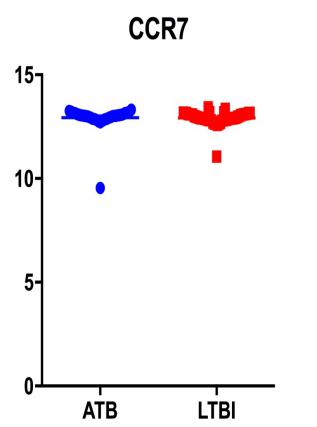

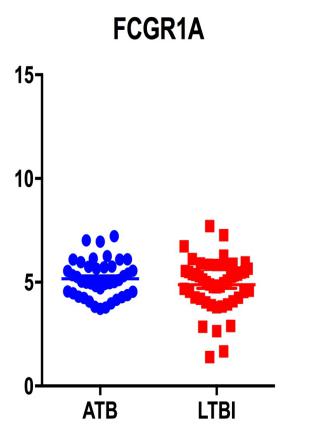

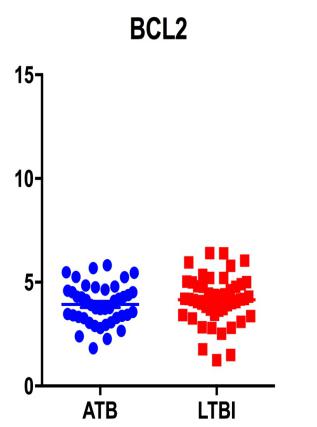

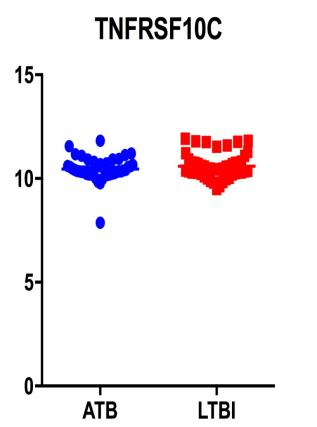

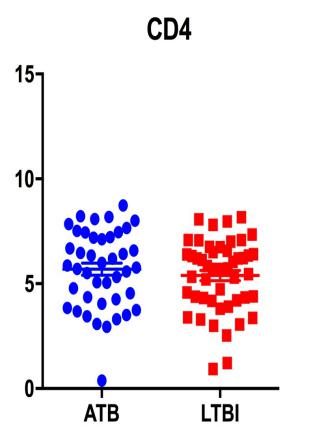


**Additional Figure S3**. The receiver operating characteristic curve (ROC) of the selected mRNA in distinguishing ATB and LTBI patients


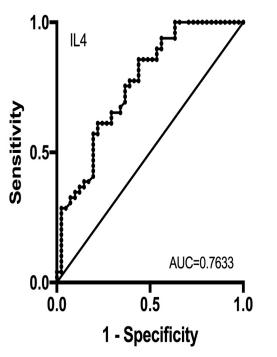

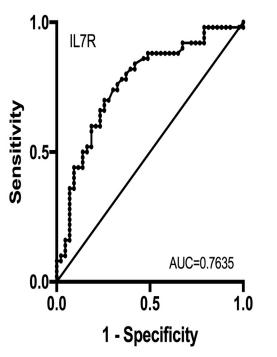

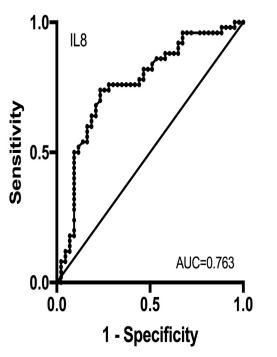

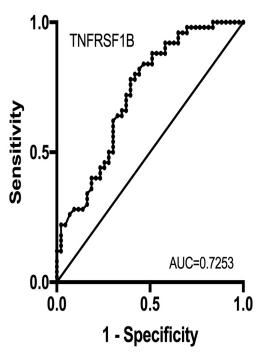

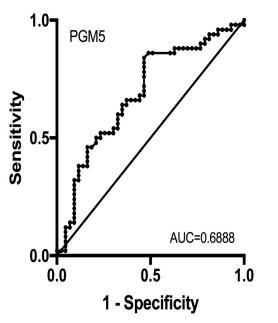

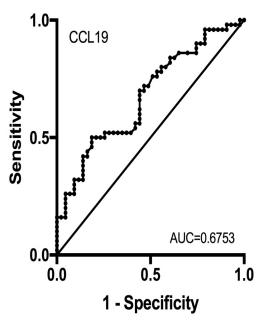

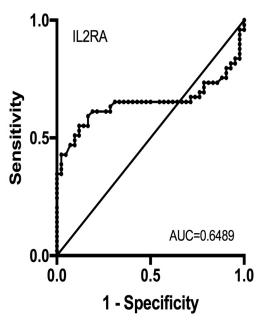

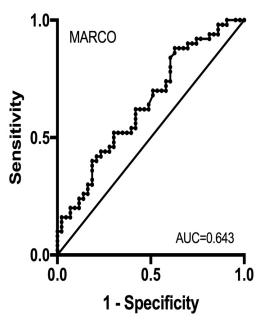

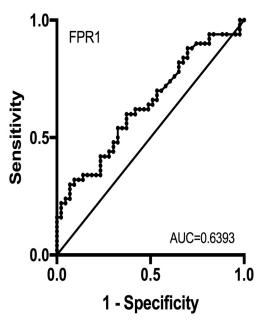

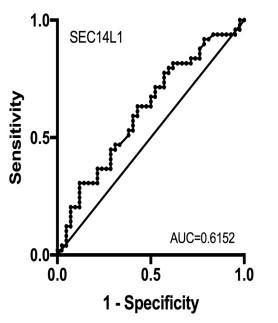

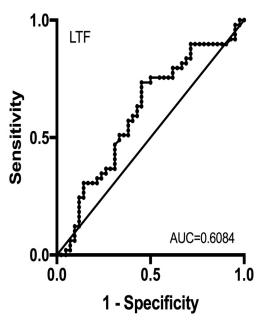

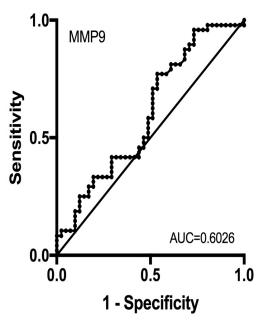

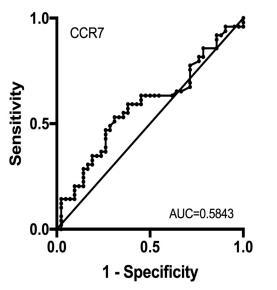

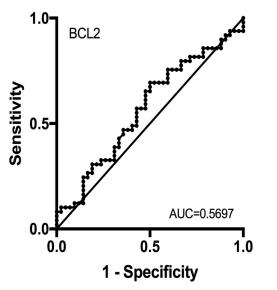

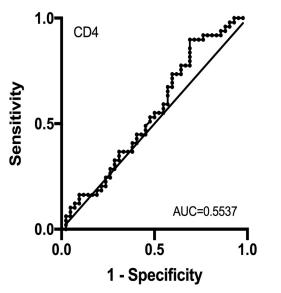

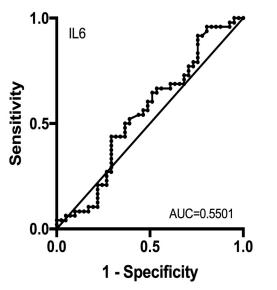

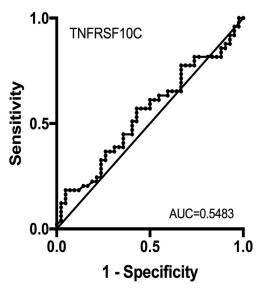

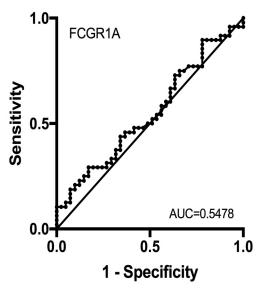

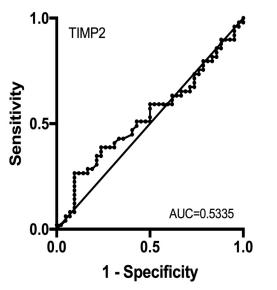

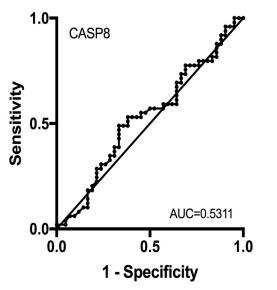

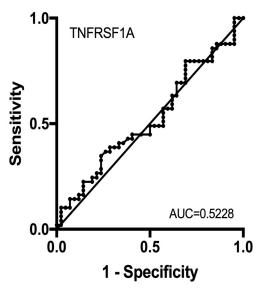

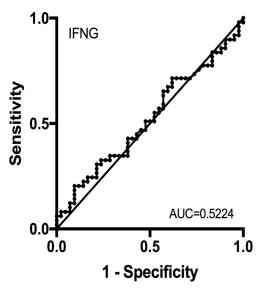

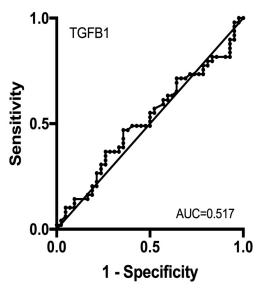

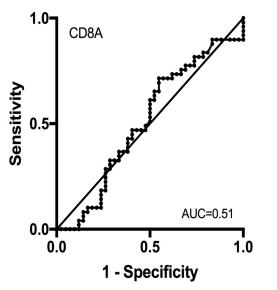

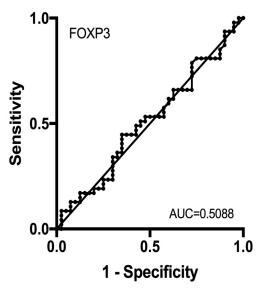

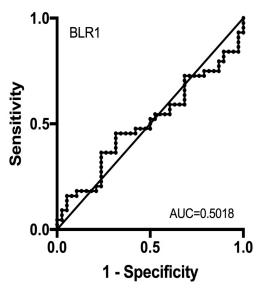

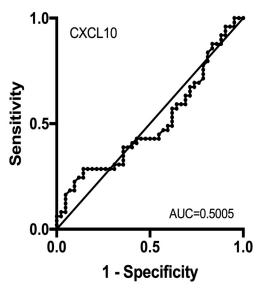

Supplement: Supplementary file 1 — Additional file 1: Table S1. The list of selected mRNAs that were reported to distinguish between ATB and LTBI in previous studies Table S2 The probes of 27 selected mRNAs for dcRT-MLPA. Table S3 The reaction steps in dcRT-MLPA. Table S4 The location and symbol of selected 107 SNPs with p value less than E-05. Figure S1. Flow chart. Figure S2. 9 mRNAs with statistically differential expression in ATB and LTBI patients. Figure S3. The receiver operating characteristic curve (ROC) of the selected mRNA in distinguishing ATB and LTBI patients. [file 12931_2020_1612_MOESM1_ESM.docx]
